# Testosterone antagonizes paraquat-induced cardiomyocyte senescence via the mIGF-1/SIRT1 signaling pathway

**DOI:** 10.1590/1414-431X20209849

**Published:** 2020-09-07

**Authors:** Xing Yu, Jianyi Zheng, Tengfei Cai, Zhijian Wang, Guiping Zhu

**Affiliations:** Cardiovascular Department, First Affiliated Hospital, Guangdong Pharmaceutical University, Guangzhou, Guangdong, China

**Keywords:** Testosterone, Paraquat, Cardiomyocyte senescence, Insulin-like growth factor-1, Sirtuin1

## Abstract

Testosterone has been demonstrated to antagonize doxorubicin-induced cardiomyocyte senescence. However, whether testosterone prevents the paraquat-induced cardiomyocyte senescence is largely unknown. The detection of SA-β-gal activity was performed using senescence β-gal staining kit and the reactive oxygen species levels were determined by reactive oxygen species assay kit. The plasmids for insulin-like growth factor 1 shRNA (sh-mIGF-1), sirtuin-1 shRNA (sh-SIRT1), scramble shRNA (sh-NC), overexpressing mIGF-1 (mIGF-1), overexpressing SIRT1 (SIRT1), and negative controls (NC) were obtained for this study. The expression of target genes was detected using quantitative real-time PCR, immunolabeling, and western blot. We found that testosterone significantly delayed the paraquat-induced HL-1 cardiomyocyte senescence as evidenced by decreasing senescence-associated β-galactosidase activity and reactive oxygen species generation, which were accompanied by the up-regulated expression of mIGF-1 and SIRT1. RNA interference to reduce mIGF-1 and SIRT1 expression showed that testosterone prevented paraquat-induced HL-1 senescence via the mIGF-1/SIRT1 signaling pathway. Furthermore, myocardial contraction was evaluated by expression of genes of the contractile proteins/enzymes, such as α-myosin heavy chain 6 (MHC6), α-myosin heavy chain 7 (MHC7), α-skeletal actin (ACTA-1), and sarco/endoplasmic reticulum calcium ATPase-2 (SERCA2). Testosterone adjusted the above four gene expressions and the adjustment was blocked by mIGF-1 or SIRT1 inhibition. Our findings suggested that the mIGF-1/SIRT1 signaling pathway mediated the protective function of testosterone against the HL-1 cardiomyocyte senescence by paraquat, which provided new clues for the mechanisms underlying the anti-aging role of testosterone in cardiomyocytes.

## Introduction

With the rapid aging of the world's population, heart failure, of which the incidence increases with age, is calling for attention (1,2). The heart undergoes a series of physiological and morphological changes with aging, which is called cardiac aging. Cardiac aging is a continuous and irreversible process, often accompanied by cardiomegaly, which is thought to contribute to myocardial dysfunction and failure (3). Therefore, the modulation of cardiomyocyte aging is of great significance to prevent the occurrence of heart failure.

Testosterone, the most important androgen, plays an important role in the cardiovascular system and its level gradually decreases with age in males (4,5). Plasma testosterone level is inversely correlated with the incidence of multiple age-related diseases in elderly men (5). Some testosterone replacement therapies have suggested that testosterone is able to improve cognitive ability (6), enhance cardiac function (7), and delay cardiomyocyte senescence (8,9). Although testosterone has multiple functions, the relationship between testosterone's effect of delaying cardiomyocyte senescence and the mIGF-1/SIRT1 pathway has not been elucidated.

Recent studies have confirmed that insulin-like growth factor 1 (IGF-1) and sirtuin-1 (SIRT1) are associated with cardiovascular aging (10-12). IGF-1 has been proven to participate in regulation of cell signaling, senescence, and apoptosis in the heart (13). Targeted administration of IGF-1 *in vivo* improves contractile function after experimental myocardial infarction via activated Akt (14). The attenuation of myocyte senescence by IGF-1 may delay the appearance of heart failure (15). Muscle restricted mIGF-1 has been reported to protect cardiomyocytes from toxic injury by inducing SIRT1 activity, whereas circulating other IGF-1 isoforms has no such affect (16). SIRT1 belongs to a nicotinamide adenine dinucleotide-dependent histone deacetylase, which plays a key role in regulating life and health (17,18), as well as delays the onset of age-dependent cardiac fibrosis and cell death (19). Ota et al. (20) found that testosterone inhibited oxidative stress-induced endothelial senescence via up-regulation of SIRT1.

Thus, we hypothesized that testosterone ameliorates cardiomyocyte senescence via the mIGF-1/SIRT1 signaling pathway. Firstly, we established the paraquat-induced cardiomyocyte senescence model to demonstrate whether testosterone protects cardiomyocytes from paraquat-induced senescence. Further studies elucidated that the mIGF-1/SIRT1 signaling pathway played an important role in the protective effect of testosterone against paraquat-induced cardiomyocyte senescence. Finally, we demonstrated that testosterone modified the gene expression of the contractile proteins/enzymes in senescent cardiomyocytes by mediating mIGF-1 and SIRT1 expression.

## Material and Methods

### Cell culture

The HL-1 mouse cardiomyocyte cell line was purchased from the Louisiana State University Medical Center (USA). Cells were cultured in Dulbecco's modified Eagle's medium (DMEM, Thermo Fisher Scientific, USA) supplemented with 5% fetal bovine serum (FBS, Life Technologies, USA), 100 U/mL penicillin (Sigma, Germany), and 100 μg/mL streptomycin (Sigma) at 37°C in a humidified 5% CO_2_ incubator (371, Thermo Fisher Scientific).

### Testosterone treatment

HL-1 cardiomyocytes were exposed to 100 μM paraquat for 1 h. After trypsin digestion, the cardiomyocytes were re-seeded in plates and cultured with DMEM containing different concentrations of testosterone (0.001, 0.01, 0.1, 1 μM). The specific cultivation time was determined in the preliminary experiment.

### SA-β-galactosidase staining

The feature of cell senescence was evaluated by enhanced senescence associated β-galactosidase (SA-β-gal) activity (21). The detection of SA-β-gal activity was performed using senescence β-gal staining kit (Beyotime, China) according to the manufacturer's instruction. Briefly, cells were rinsed twice with phosphate-buffered saline (PBS), fixed with 2% formaldehyde/0.2% glutaraldehyde for 15 min at room temperature and incubated with SA-β-gal staining solution for 12 h at 37°C in humidified incubator without CO_2_. The cells were observed under fluorescence microscope (MF52, Mshot, China).

### Determination of reactive oxygen species

Studies have shown that the specific increase of reactive oxygen species (ROS) levels is a key to the inducing and maintaining the cellular senescence process (22). The ROS levels were determined by reactive oxygen species assay kit (Beyotime) according to the manufacturer's instruction. Briefly, HL-1 cells were seeded in culture plates at a density of 1×10^5^/well. After attachment, cells were washed once with PBS and incubated with 10 μM DCFH-DA at 37°C for 20 min. Subsequently, the excess probe was rinsed three times with PBS to ensure that only intracellular ROS would be measured. Finally, the DCF florescence distribution of 2,000 cells was detected by a BD Accuri C6 flow cytometer (BD Biosciences, USA). The results were analyzed using FlowJo data analysis software (USA).

### Quantitative real-time PCR analysis

As for mRNA detection, total RNA was extracted from treated HL-1 cells using TRIzol Reagent (Qiagen, Germany) according to the instructions, and reversed transcribed to cDNA using PrimeScript™ RT reagent kit with gDNA Eraser (Takara, China). Gene expression levels were performed on an ABI Prism 7500 system (Applied Biosystems, USA) using One Step SYBR^®^ PrimeScript^®^ PLUS RT-PCR Kit (Takara), with each sample prepared in triplicate according to the manufacturer's recommendation. β-actin mRNA was analyzed simultaneously as a control and used for normalization of data. The relative expression of the mRNA was quantified using the 2^-ΔΔCt^ method. The sequences of primer pairs are listed in Table 1.

**Table 1:** Sequences of all primer pairs in the study.

Gene name	Forward primer (5′-3)	Reverse primer (5′-3′)
mIGF-1	CGGCAGGAGACATTTGATTTG	TCTTTCTCCTCTCTCCCTTCTT
SIRT1	GTAAGCGGCTTGAGGGTAAT	GTTACTGCCACAGGAACTAGAG
β-actin	GAGGTATCCTGACCCTGAAGTA	CACACGCAGCTCATTGTAGA
ACTA-1	GAGGTATCCTGACCCTGAAGTA	CACACGCAGCTCATTGTAGA
MHC6	CACTTCTCCTTGGTCCACTATG	GGGAGGACTTCTGGTACAAAC
MHC7	CCATCTCTGACAACGCCTATC	GGATGACCCTCTTAGTGTTGAC
SERCA2	TACCTGGCTATTGGCTGTTATG	GGAAATGACTCAGCTGGTAGAA

### Plasmid construction and cell transfection

The plasmids for mIGF-1 shRNA (sh-mIGF-1), SIRT1 shRNA (sh-SIRT1), scramble shRNA (sh-NC), overexpressing mIGF-1 (mIGF-1), overexpressing SIRT1 (SIRT1), and negative controls (NC) were obtained from Gene Denovo Biotechnology Company (China). The sequence of sh-mIGF-1, sh-SIRT1, and sh-NC are shown in Table 2. The HL-1 cells were transfected with overexpressing RNAs and shRNAs plasmids using lipid-based reagent lipofectamineTM 2000 (Invitrogen) according to the manufacturer's instruction. At 48 h post transfection, transfection efficiency was verified by quantitative real-time PCR to determine the relative expression levels of mIGF-1 or SIRT1. As shown in Supplementary Figure S1B and C, the mIGF-1 and SIRT1 overexpression vectors were successfully constructed and transfected, which were used in the study. As shown Supplementary Figure S1D and E, the efficiency of shRNAs to knock down corresponding RNA expression was tested, and the most efficient shRNAs (sh-mIGF-1 2 and sh-SIRT1 3) were used in the study.

**Table 2:** Sequence of sh-mIGF-1, sh-SIRT1, and sh-NC mRNA.

Name	shRNA sequence
sh-mIGF-1	ACCGGGCACCTGCAATAAAGATACACATCATACTCGAGTATGATGTGTATCTTTATTGCAGGTGCTTTTTTGAATTC
sh-SIRT1	ACCGGGATGCTGTGAAGTTACTGCTACTCGAGTAGCAGTAACTTCACAGCATCTTTTTTGAATTC
sh-NC	ACCGGCCTAAGGTTAAGTCGCCCTCGCTGAGCGAGGGCGACTTAACCTTAGGTTTTTGAATTC

### Western blot analysis

Cells were lysed in radio immunoprecipitation assay (RIPA) buffer (Vazyme, China) supplemented with protease inhibitor cocktail (Roche, Switzerland) on ice for 15 min, followed by centrifugation (12000 *g* for 5 min at 4°C) to remove cell debris. Protein concentrations were determined by BCA Protein Assay kit (Thermo Fisher Scientific) according to the manufacturer's instruction. Equal mass of total protein was separated by 10% SDS-PAGE (Vazyme), and then transferred to a polyvinylidene difluoride (PVDF) membrane (Invitrogen). The PVDF membranes were blocked with 5% skim milk (BD Biosciences) at room temperature for 1 h, rinsed, and then incubated overnight at 4°C with the following primary antibodies: mIGF-I (1:1,500 dilution, Cell Signaling Technology, USA) and SIRT1 (1:1,000 dilution, Cell Signaling Technology). After removing the primary antibody, the membranes were incubated at room temperature for 30 min with horseradish peroxidase-conjugated secondary antibody (1:1000 dilution, Cell Signaling Technology). The protein blots were detected using a high signal ECL western blotting substrate (Tanon, China). Protein expression levels were quantified by densitometry using ImageJ software (National Institutes of Health, USA) and normalized to β-actin.

### Immunofluorescence assay

At indicated times, cells were fixed with pre-cooled 4% paraformaldehyde (Sigma) for 15 min, washed with PBS, and permeated with 0.5% Triton X-100 (Sigma) for 20 min at room temperature. After blocking with 5% bovine serum albumin (BSA, Sigma) for 30 min, cells were incubated with the following primary antibody overnight at 4°C: mIGF-1 (1:500 dilution, Cell Signaling Technology) and SIRT1(1:500 dilution, Cell Signaling Technology), and then with the diluted fluorophore-conjugated secondary antibody (Abcam, USA) for 1 h at room temperature. The cells were stained with DAPI (Abcam) according to the manufacturer's instruction. Finally, images were captured using a fluorescence microscope with a digital camera (MF52, Mshot) and analyzed with Image-Pro Plus version 6.0 software (Media Cybernetics, USA).

### Statistical analysis

Data are reported as means±SE. Analysis was performed using one-way ANOVA followed by Dunnett's multiple comparisons test, and P<0.05 was considered as significant. All statistical analyses were performed using the Graphpad Prism 7.0 software.

## Results

### Testosterone protected cardiomyocytes against PQ-induced senescence

Firstly, we assessed the effects of paraquat (1,1-dimethyl-4,4-bipyridilium dichloride, PQ, 100 μM) treatment for 24, 48, and 72 h on cell senescence in HL-1 cardiomyocytes by staining for SA-β-gal. The specific data of PQ-induced cell senescence in preliminary experiments is shown in Table 3. The ratio of senescent cells increased in a time-dependent manner, and was significantly increased in response to 100 μM PQ for 72 h (Supplementary Figure S1A). As a result, PQ (100 μM, 72 h) was chosen as the optimal conditions for the subsequent studies.

**Table 3:** Comparison of cell senescence rates of the control (CK) and paraquat (PQ) groups.

Group	Senescence rates (means±SE)	Group	Senescence rates (means±SE)	P value (CK *vs* PQ)
CK-24h	9.09±1.60	PQ-24h	12.41±0.79	0.119
CK-48h	17.29±1.30	PQ-48h	19.05±1.70	0.584
CK-72h	26.67±1.60	PQ-72h	30.88±1.11	0.008

Each group of cells was counted in 4 fields randomly selected under the microscope. Cell senescence rate (%): Number of senescent cells/total number of cells × 100%. Student's *t*-test.

**Figure 1:**
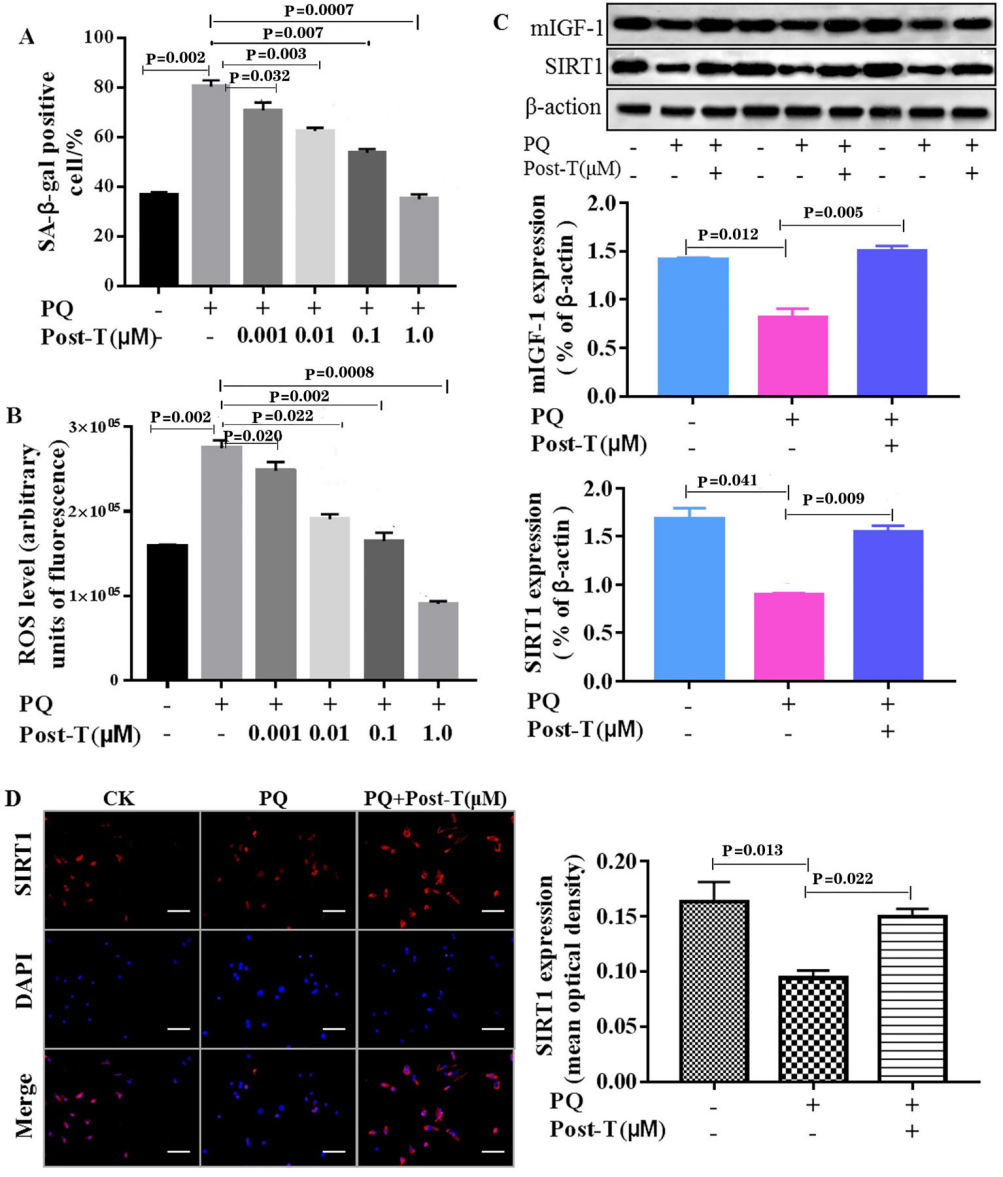
Testosterone (T) prevents paraquat (PQ)-induced cell senescence in HL-1 cardiomyocytes. **A**, Percentages of senescence-associated β-galactosidase (SA-β-gal)-positive HL-1 cells after no treatment or exposure to PQ for 1 h, and with or without incubation with testosterone for 72 h at the indicated concentrations. **B**, Effect of testosterone on reactive oxygen species (ROS) production of HL-1 cardiomyocytes after the same treatments as in panel A. **C**, Protein expression of mIGF-1 and SIRT1 in HL-1 cardiomyocytes was measured by western blotting after no treatment or exposure to PQ for 1 h post-incubated with or without testosterone (1.0 μM) for 72 h. **D**, Detection of SIRT1 expression in HL-1 cardiomyocytes by immunofluorescence after the same treatment as in panel C. Scale bar indicates 50 μm. Data are reported as means±SE of four independent experiments (ANOVA and Dunnett's multiple comparisons test).

To investigate the role of testosterone on PQ-induced HL-1 cardiomyocyte senescence, HL-1 cardiomyocytes were treated with different concentrations of testosterone (0.001, 0.01, 0.1, and 1.0 μM) in the presence of PQ for 72 h. The results showed that testosterone inhibited PQ-induced HL-1 cell senescence in a concentration-dependent manner, as evidenced by the decreasing ratio of SA-β-gal positive cells as well as ROS level (Figure 1A and B). Interestingly, the ratio of SA-β-gal positive cells and the ROS level in testosterone (1.0 μM) plus PQ-treated HL-1 cells were significantly decreased compared with those treated with PQ alone, but there was no difference with the untreated cells. Therefore, testosterone (1.0 μM) was chosen as the optimal concentration for the subsequent studies.

### Testosterone promoted protein expression of mIGF-1 and SIRT1 in PQ-treated cardiomyocytes

Previous studies have demonstrated that locally acting mIGF-1 and SIRT1 play a role in the regulation of organism health-span (12,23). Thus, we further investigated whether testosterone affects the expression of mIGF-1 and SIRT1 in PQ-treated HL-1 cardiomyocytes. Firstly, we used western blot assay to detect the protein levels of mIGF-1 and SIRT1 in HL-1 cardiomyocytes with or without testosterone (Figure 1C). Compared with the non-treatment groups, the expression of mIGF-1 and SIRT1 decreased significantly in PQ-treatment groups, and increased significantly in PQ plus testosterone treatment groups. Moreover, immunofluorescence assay was performed to point out the localization of SIRT1 in HL-1 cardiomyocytes (Figure 1D). The data revealed that SIRT1 (red) was mostly located inside the nuclei (blue). Paraquat significantly decreased the expression of SIRT1 in HL-1 cardiomyocytes, as the mean fluorescent density reduced, while testosterone plus PQ treatment significantly increased the expression. Taken together, these results suggested that testosterone promoted the expression of mIGF-1 and SIRT1 in cardiomyocytes.

### Testosterone inhibited PQ-induced senescence via activating mIGF-1 expression

**Figure 2:**
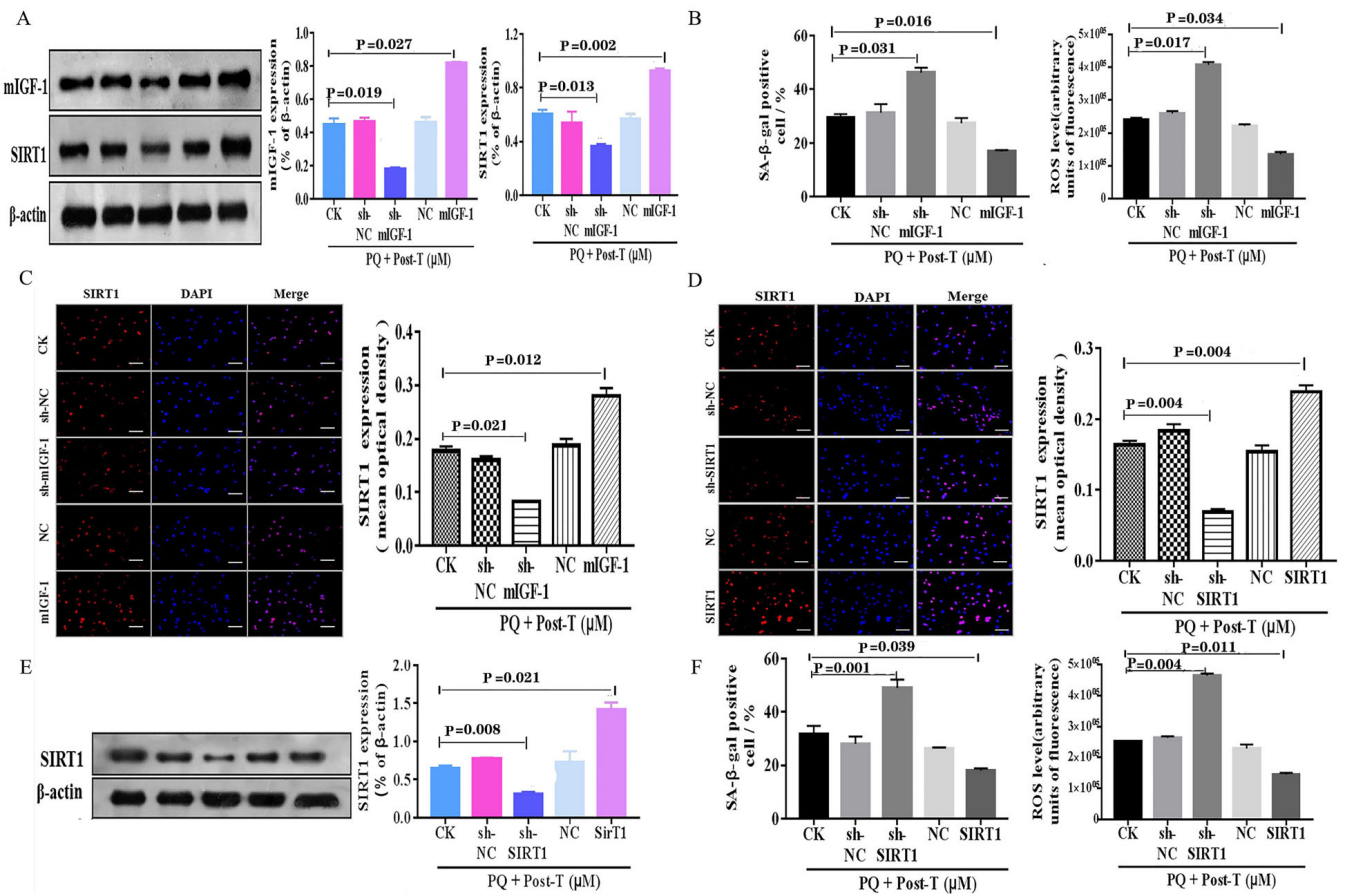
Testosterone (T) delayed paraquat (PQ)-induced cardiomyocyte senescence via the mIGF-1/SIRT1 pathway. **A**, Representative western blotting and band densitometry for mIGF-1 and SIRT1 in HL-1 cardiomyocytes transfected with or without sh-mIGF-1 and mIGF-1 in the presence of PQ and T. **B**, Percentages of senescence-associated β-galactosidase (SA-β-gal)-positive HL-1 cardiomyocytes and reactive oxygen species (ROS) production of cardiomyocytes after the same treatments as in panel A. **C**, Detection of SIRT1 expression in HL-1 cardiomyocytes by immunofluorescence and the statistical results after the same treatment as in panel A. Scale bar indicates 50 μm. **D**, Detection of SIRT1 expression in HL-1 cardiomyocytes by immunofluorescence and the statistical results after transfected with or without sh-SIRT1 and SIRT1 in the presence of PQ and T. Scale bar indicates 50 μm. **E**, Representative western blotting and band densitometry for SIRT1 in HL-1 cardiomyocytes after the same treatment as in panel 2D. **F**, Percentages of SA-β-gal-positive HL-1 cardiomyocytes and the ROS production of cardiomyocytes after the same treatments as in panel 2D. Data are reported as means±SE of four independent experiments (ANOVA and Dunnett's multiple comparisons test). CK: control.

To further investigate whether the protective effect of testosterone against PQ-induced senescence was associated with mIGF-1 expression level, we first used western blot assay to detect the protein level of mIGF-1 in HL-1 cardiomyocytes transfected with mIGF-1 and sh-mIGF-1 (Figure 2A). Under PQ plus testosterone condition, the protein expression of mIGF-1 in HL-1 cardiomyocytes transfected with mIGF-1 was significantly increased compared to that in no transfection, and significantly reduced in HL-1 cardiomyocytes transfected with si-mIGF-1. To further validate the role of mIGF-1 in testosterone-delayed cell senescence, the SA-β-gal assay and ROS level were assessed under the overexpression and inhibition of mIGF-1condition induced by mIGF-1 or si-mIGF-1 (Figure 2B). The results showed that SA-β-gal positive cell and ROS levels were significantly decreased in HL-1 cardiomyocytes transfected with mIGF-1 compared to that in no transfection, while significantly increased in cardiomyocytes transfected with si-mIGF-1. Taken together, these data indicated that testosterone protected HL-1 cardiomyocytes against PQ-induced senescence via up-regulating mIGF-1 expression.

### Testosterone mediated SIRT1 activity to protect HL-1 cardiomyocytes against PQ-induced senescence

To explore the underlying mechanism by which testosterone activates mIGF-1 in PQ-induced HL-1 cardiomyocytes, we first evaluated the effect of mIGF-1 overexpression or inhibition on SIRT1 expression in PQ-induced HL-1 cardiomyocytes treated with testosterone. We used western blot assay to detect the protein levels of SIRT1 in HL-1 cardiomyocytes transfected with mIGF-1 and sh-mIGF-1. The results showed that the expression of SIRT1 significantly increased in the testosterone plus mIGF-1 group compared to that in the testosterone group, and significantly decreased in the testosterone plus sh-mIGF-1 group (Figure 2A). In addition, analysis of the location and expression levels of SIRT1 were measured by immunofluorescence assay, and the results revealed that SIRT1 (red) was located inside the nuclei (blue), and the expression levels of SIRT1 in each group were consistent with western blot analysis (Figure 2C). The above data indicated that the up-regulation of SIRT1 expression was regulated by mIGF-1.

Since SIRT1 changes are in line with mIGF-1 activation, we assessed whether SIRT1 played roles in testosterone-delayed cardiomyocyte senescence by silencing or overexpressing SIRT1. We first measured the transfection efficiency by immunofluorescence assay and western blotting (Figure 2D and E). The results showed that SIRT1 transfection significantly increased the SIRT1 expression in the presence of PQ plus testosterone, but sh-SIRT1 transfection significantly decreased the SIRT1 expression. Moreover, under PQ plus testosterone condition, SIRT1 overexpression accelerated the protective effect of testosterone on PQ-induced HL-1 cardiomyocyte senescence, while silencing SIRT1 triggered a significant increase in SA-β-gal positive cell and ROS levels compared to the control group (Figure 2F). In summary, these data demonstrated that testosterone regulated the activity of SIRT1 to protect HL-1 cardiomyocytes from PQ-induced senescence.

### Testosterone-activated mIGF-1/SIRT1 pathway-regulated gene expression of contractile proteins/enzymes in cardiomyocyte senescence

**Figure 3:**
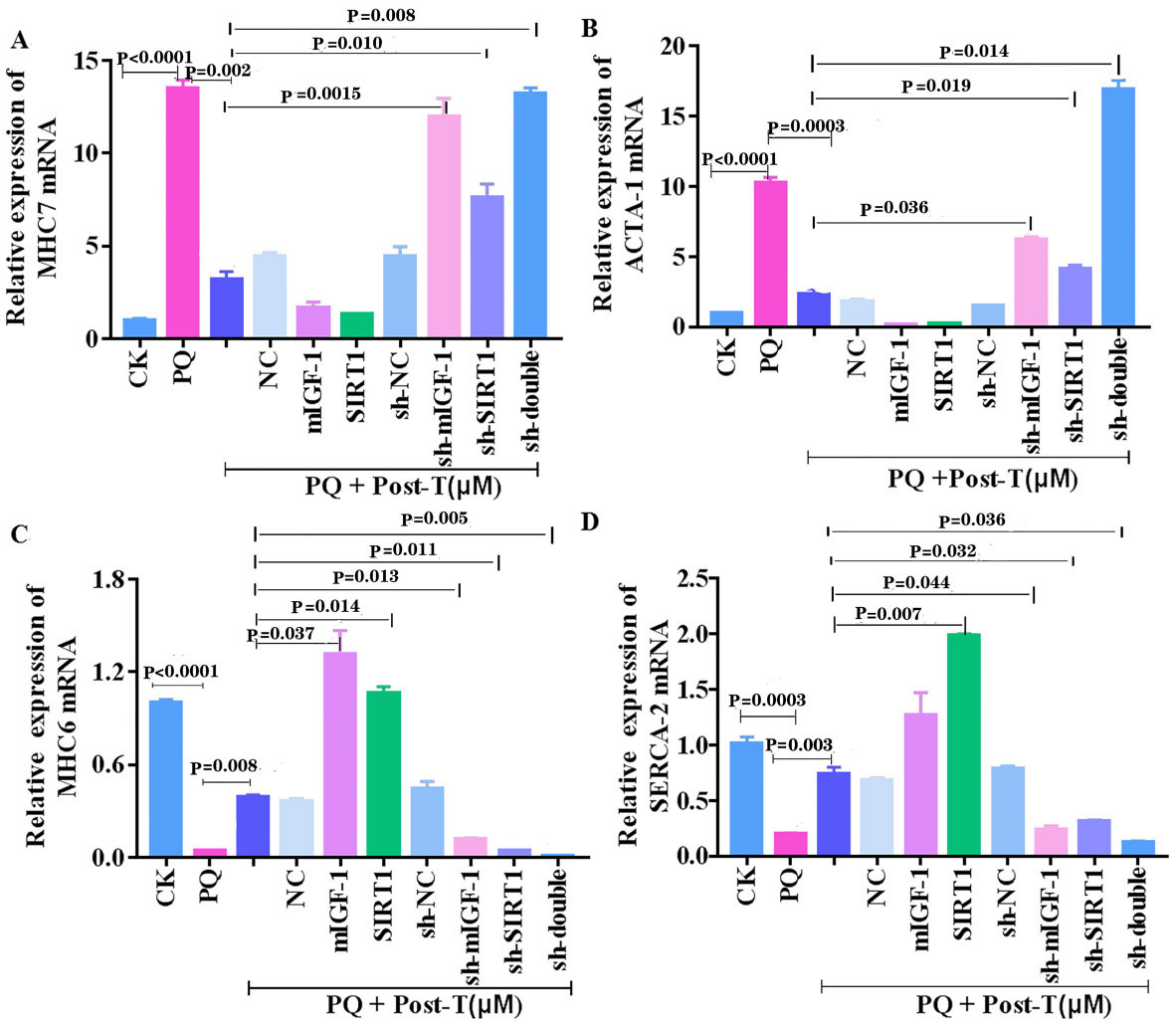
Testosterone (T) changed the gene expression of the contractile protein/enzyme via mediating mIGF-1/SIRT1 pathway in HL-1 cardiomyocytes. The expressions of MHC7 mRNAs (**A**), ACTA-1 mRNAs (**B**), MHC6 mRNAs (**C**), and SERCA2 mRNAs (**D**) were examined by qRT-PCR. Data are reported as means±SE of four independent experiments (ANOVA and Dunnett's multiple comparisons test). PQ: paraquat; CK: control; NC: negative control.

Cardiomyocyte aging leads to the increase of myocardial stiffness and the decrease of systolic functions, which are related to gene expression of the contractile proteins/enzymes, including α-myosin heavy chain 6 (MHC6) conversion to α-myosin heavy chain 7 (MHC7), upregulating α-skeletal actin (ACTA-1), and downregulating sarco/endoplasmic reticulum calcium ATPase-2 (SERCA2) (16,24). As shown in Figure 3, PQ significantly increased the expression of ACTA1 and MHC7 and decreased the expression of SERCA2 and MHC6 in HL-1 cardiomyocytes, and these changes were abolished by testosterone. Interestingly, the inhibition of the mIGF-1 and SIRT1 expression in PQ-induced HL-1 cardiomyocyte treated with testosterone significantly increased the expressions of ACTA1 and MHC7 but decreased the expressions of SERCA2 and MHC6 compared with testosterone alone. In brief, these results suggested that testosterone adjusted the gene expression of the contractile proteins/enzymes in senescent cardiomyocytes via mediating mIGF-1/ SIRT1 signaling pathway.

## Discussion

In the present study, we confirmed that testosterone prevented PQ-induced cardiomyocyte senescence, and further studies indicated that the protection was by regulating gene expression of the contractile proteins/enzymes via mIGF-1/SIRT1 signaling pathway.

Although PQ is widely used in agricultural practices as a quaternary nitrogen herbicide, it is extremely toxic to human beings, animals, and insects, for which there is no specific antidote (25), leading to acute lung and heart damage (26,27). In particular, current studies have shown that PQ treatment induces myocardial dysfunction (28-30) and senescence (31). The idea of PQ-induced cardiomyocyte senescence is further supported by data from the present study. Our results also demonstrated that PQ treatment for 72 h induced senescence-related phenotypes in HL-1 cells, including the increase of SA-β-gal activity and ROS generation. These findings are consistent with previously reported phenotypes associated with cardiomyocyte senescence (31).

Testosterone is the major systemic androgen and its level gradually decreased with age in men (4). Studies have shown that a low level of testosterone is closely related to the development of aging-associated cardiovascular disease, and testosterone replacement therapies play a beneficial role in male cardiovascular disease (32,33). Zhang et al. (9,34) found that testosterone deficiency induces cardiomyocyte senescence, and physiological testosterone therapies delay cardiomyocyte senescence via an AR-independent pathway. In addition, testosterone antagonizes doxorubicin-induced cardiomyocyte senescence by modulating telomere binding factor 2 (9).

IGF-1 and SIRT1 are novel important mediators of cell homeostasis and cardiac stress that have been connected to the lifespan regulation in multiple organisms (23,35,36). Studies have revealed that circulating levels of IGF-1 significantly decline with age both in elderly human and experimental animals, and the age-related decline in circulating IGF-1 is causally implicated in the development of aging phenotype in the heart and vasculature (12,37,38). Moreover, cardiac-specific overexpression of IGF-1 protects the heart from PQ-induced oxidative stress and lethality via activating SIRT1 expression (16). In addition, SIRT1 belongs to the sirtuin family of nicotinamide adenine dinucleotide (NAD)-dependent protein deacetylases, which is a crucial protective molecule against cardiovascular aging (10). SIRT1 overexpression or activation mediates anti-aging and cell-protective effects in the heart *in vivo* (19). Moreover, an age-mediated decrease in SIRT1 activity was observed in the heart of aged rat (39). These studies suggest that IGF-1 and SIRT1 play an important role in suppressing the aging process of organs and cells. Our results are consistent with those studies demonstrating that IGF-1 and SIRT1 levels were significantly down-regulated in PQ-induced cardiomyocyte aging. However, it is unclear whether testosterone prevents PQ-induced cardiomyocyte aging associated with activation of the IGF-1/SIRT1 signaling pathway. In the present study, our results indicated that testosterone intervention blocked PQ-induced HL-1 cell aging-related phenotypes, which were accompanied by up-regulation of IGF-1 and SIRT1 proteins. Importantly, inhibition of IGF-1 and SIRT1 significantly exacerbated senescence-related phenotypes including increased SA-β-gal activity and ROS production in PQ-induced HL-1 cells with testosterone intervention, which were accompanied by the up-regulation of ACTA-1 and MHC-7 and the down-regulation of MHC-6 and SERCA2. Therefore, it was reasonable to conclude that IGF-1 and SIRT1 were at least in part responsible for testosterone-mediated protection against the cardiomyocyte aging induced by PQ. Previous studies show that SIRT1 is a downstream mediator of mIGF-1 action in the heart, and mIGF-1 protects cardiomyocytes from oxidative stress via SIRT1 activity *in vitro* and *in vivo* (16). Furthermore, cardiac-restricted mIGF-1 transgene induced systemic changes in a SIRT1-dependent manner, such as high blood pressure, leukocytosis, and an enhanced fear response (40). In the present study, our results revealed that mIGF-1 inhibition blocked SIRT1 expression/activity in PQ-induced HL-1 cells with testosterone, suggesting that testosterone activated SIRT1 via mIGF-1 to delay cardiomyocyte senescence caused by PQ.

There were limitations in our study: 1) the HL-1 cardiomyocyte cell line was used as the test object, and to a certain extent, it could not completely simulate the characteristics of mouse primary cardiomyocytes; 2) the effect of testosterone on aging of cardiomyocytes in our study was dose-dependent, but the dose threshold of this benefit was not proven. Therefore, an intervention with a higher dose group could provide a clearer understanding of this problem; and 3) it was not clear whether there was an internal mechanism molecule between mIGF-1 and SIRT1. Future experiments should be developed to further clarify this issue.

In summary, our data demonstrated that the protective role of testosterone in PQ-induced cardiomyocyte aging was attributed to mediating the mIGF-1/SIRT1 signaling pathway, thereby suppressing cardiac aging-associated phenotypes and modifying contractile protein/enzyme expression. Further studies are necessary to verify whether testosterone also functions in inhibition of cardiac aging via the mIGF-1/SIRT1 pathway in primary cardiomyocytes and animal models, as well as to explore the downstream signaling pathway of testosterone-regulated SIRT1 involved in anti-cardiac aging.

## Supplementary Material

Click here to view [pdf].
